# Successful Extracorporeal Membrane Oxygenation Implementation at a Non-tertiary Medical Center: A Single-Center Experience

**DOI:** 10.7759/cureus.46982

**Published:** 2023-10-13

**Authors:** Brian Hassani, Trung Tran, Prakruti Hansaliya, Wes Kelley, Ronnie Enfinger, Danna Nelson, Lauren Wright

**Affiliations:** 1 Critical Care, Baptist Medical Center South, Montgomery, USA; 2 Cardiothoracic Surgery, Mercy Hospital Northwest Arkansas, Rogers, USA; 3 Cardiothoracic Surgery, Baptist Medical Center South, Montgomery, USA; 4 Pharmacy, Baptist Medical Center South, Montgomery, USA

**Keywords:** ecmo outcome, community hospital, implementation, development, extracorporeal membrane oxygenation

## Abstract

The inexperience and limited resources at non-tertiary medical centers pose unique challenges to the successful development of an extracorporeal membrane oxygenation (ECMO) program. The current literature does not provide a detailed framework that addresses the unique challenges encountered at these facilities.

We outline a proactive approach to developing an ECMO program and provide a retrospective analysis of patient demographics, clinical characteristics, ECMO configuration, duration of ECMO support, major adverse events, and survival to hospital discharge. Data are summarized using mean, median, percentages, standard deviation, and interquartile range.

Eleven patients were cannulated between December 2021 to March 2023. The age range of the patients who received ECMO varied significantly, with the youngest being 25 years old and the oldest being 69 years old. The mean age was 38 years old, with a standard deviation of 15.9. Hypertension was the most common co-morbid condition occurring in 64% (n=7) of patients. Only one patient had a major adverse event, and survival to hospital discharge was 73% (n=8). Of the patients that survived hospital discharge, seven patients were discharged home and one to a rehabilitation facility.

These findings suggest that the safe implementation of an ECMO program at a non-tertiary hospital with inexperienced staff and limited resources is feasible. Adherence to established guidelines is essential for new programs, especially with regard to patient selection. Furthermore, a proactive approach that emphasizes high-yield training techniques, patient management protocols, and strategies that mitigate adverse events may be the key to achieving survival rates that exceed those of larger academic hospitals.

## Introduction

The influenza A (H1N1) epidemic and the COVID-19 pandemic have highlighted the importance of extracorporeal membrane oxygenation (ECMO) in critical care [1-3]. Venovenous (VV) and venoarterial (VA) ECMO have become mainstream rescue strategies for refractory respiratory failure and cardiogenic shock, with a fivefold increase in ECMO runs over the last decade [4]. Despite being a potentially life-saving therapy, there are limited non-tertiary hospitals listed as ECMO centers by the Extra Corporeal Life Support Organization (ELSO) [5]. While tertiary medical centers with higher volumes and more experience have been associated with lower mortality rates, patients are often too unstable for transport to these facilities without ECMO support [6]. Regional referral centers with specialized transport teams that have remote cannulation capabilities may seemingly be a potential solution. However, limited bed availability at the receiving facility or the need for emergent cannulation makes utilization of these programs difficult at times.

The COVID-19 pandemic exposed the weaknesses of healthcare systems, leading to staffing shortages and limited bed availability. Given these challenges, timely transport to experienced ECMO centers may not be possible. It may be necessary for smaller community hospitals to consider developing an ECMO program within a hub-and-spoke regional network to ensure timely access to patients.

The current literature focuses on larger medical centers and only provides general guidance for the development of an ECMO program [7-14]. Many non-tertiary hospitals lack the resources and experience of larger centers necessitating a more tailored framework for ECMO program development.

Baptist Medical Center South, a regional referral center in Montgomery, Alabama, began the planning phase of a nurse-driven ECMO program in January 2021 after having difficulty transferring patients to ECMO centers during the initial waves of the COVID-19 pandemic. The program became operational in December 2021. To complement the current literature, the approach utilized to develop the program and patient outcomes during the first 14 months is described.

## Materials and methods

The authors sought to describe an approach to program design, protocol development, cannulation, patient management, and staff training. A retrospective analysis was conducted on all patients treated between December 2021 and March 2023, evaluating patient demographics, clinical characteristics, cannulation configuration, duration of ECMO support, major adverse events, and in-hospital mortality. 

Major adverse events are defined as major hemorrhage, limb ischemia, accidental decannulation, vessel perforation or laceration, and cardiac arrest secondary to ECMO circuit failure. Major hemorrhage is defined as intracranial hemorrhage or hemorrhage requiring two or more units of packed RBCs in 24 hours. Data are summarized using mean, percentages, standard deviation, and interquartile range. Statistical calculations were performed using Microsoft Excel and Statistical Product and Service Solutions (SPSS) (IBM SPSS Statistics for Windows, Armonk, NY) data analysis software.

Program design and structure

The first step in the planning phase of the program was to establish administrative commitment and staff dedication. It was crucial to have both financial support and a team that was willing to learn and adapt to challenges before moving forward with the program. Once this was established, Baptist South committed approximately 200,000 dollars to purchase four ECMO circuits, including oxygenators, eight arterial cannulas, and 12 venous cannulas.

Based on the high-risk nature of ECMO, especially with inexperienced staff, the program was developed in a stepwise fashion to provide safe and effective ECMO care. To achieve these objectives, ECMO management was simplified, and bedside nurses were provided with extra support during the early phases of the program. As part of this effort, the LifeSPARC circuit by LivaNova was acquired for its ease of use and reliable clinical consultants who offer ECMO education and in-person bedside assistance. Moreover, the decision was made to exclusively implement VV ECMO during the first six months of the program. The rationale for this approach was to build staff confidence, experience, and knowledge before managing more complex VA ECMO patients.

Program leadership includes an intensivist with prior ECMO experience designated as the ECMO program director, a cardiothoracic surgeon, an ECMO coordinator, two perfusionists, and a cardiovascular intensive care unit nurse (CVICU) manager. The cardiothoracic surgeon provides surgical backup and performs cannulations in the operating room if necessary. Equipment procurement falls under the purview of two perfusionists who developed an ECMO cart and ensure it remains ready for deployment. They also round daily on all ECMO patients to assess circuit integrity and functionality. The CVICU nurse manager is responsible for staffing ECMO patients with two experienced critical care nurses: a bedside nurse and an ECMO nurse. The ECMO nurse has been extensively trained and cleared by the ECMO medical director to oversee the care of ECMO patients and manage the ECMO circuit. The bedside nurse assists with routine tasks associated with the care of critically ill patients.

Referrals and cannulation

**Figure 1:**
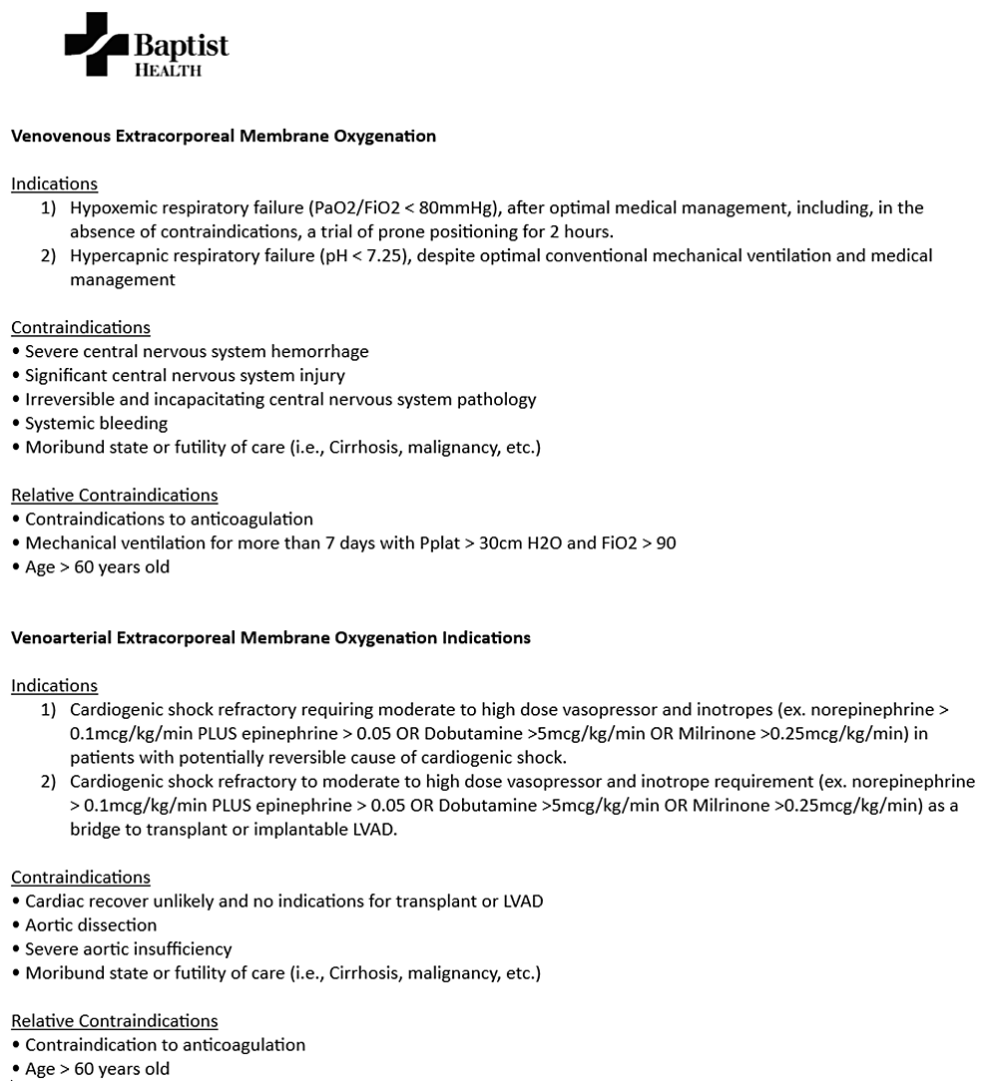
Extracorporeal Membrane Oxygenation (ECMO) Cannulation Criteria mmHg: millimeters of mercury, Pplat: plateau pressure, LVAD: left ventricular assist device

**Figure 2:**
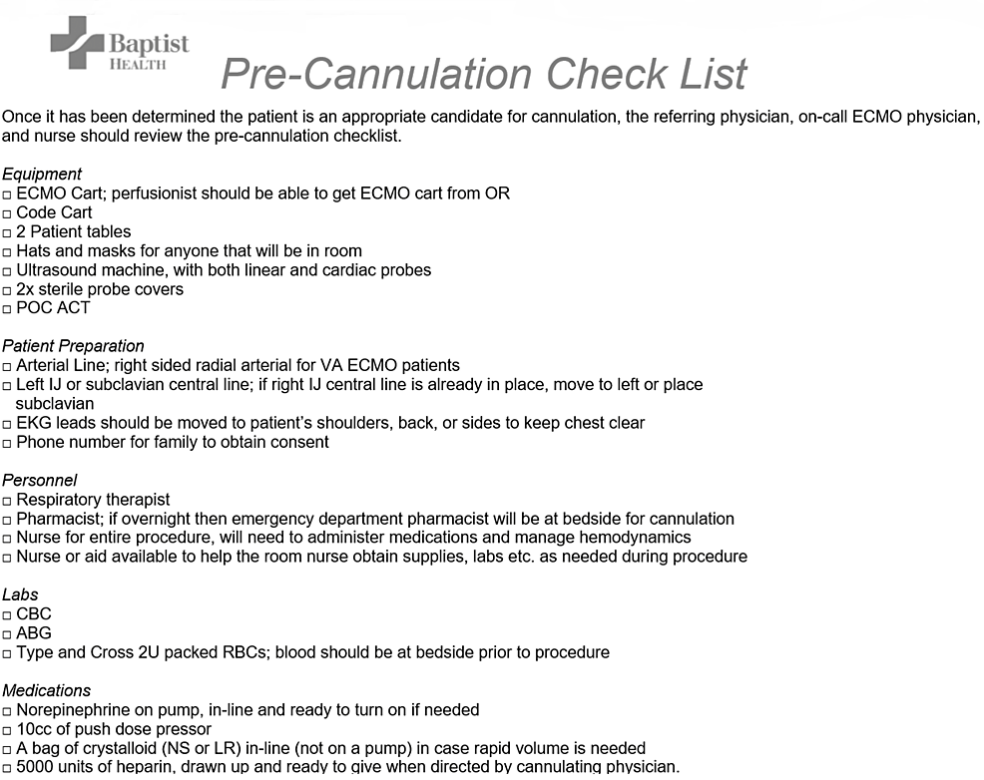
Pre-cannulation Check List OR: operating room, POC: point of care, ACT: activated clotting time, IJ: internal jugular, EKG: electrocardiogram, CBC: complete blood count, ABG: arterial blood gas, RBCs: red blood cells, NS: normal saline, LR: lactate ringer

All ECMO consults are referred to the ECMO program director, who determines ECMO candidacy in accordance with major ECMO trials and ELSO recommendations (Figure 1) [15,16]. For VA ECMO cases, the program director discusses the case with cardiothoracic surgery prior to cannulation. Once it is determined that ECMO is indicated, the ECMO team consisting of a perfusionist and cardiovascular surgery team is mobilized by the ECMO coordinator. Meanwhile, the ECMO nurse along with the referring and cannulating physicians reviews the pre-cannulation checklist in preparation for cannulation (Figure 2). Whenever possible, peripheral cannulations are performed at the bedside under ultrasound guidance by the ECMO program director or cardiothoracic surgeon to minimize the risks associated with transport.

**Figure 3:**
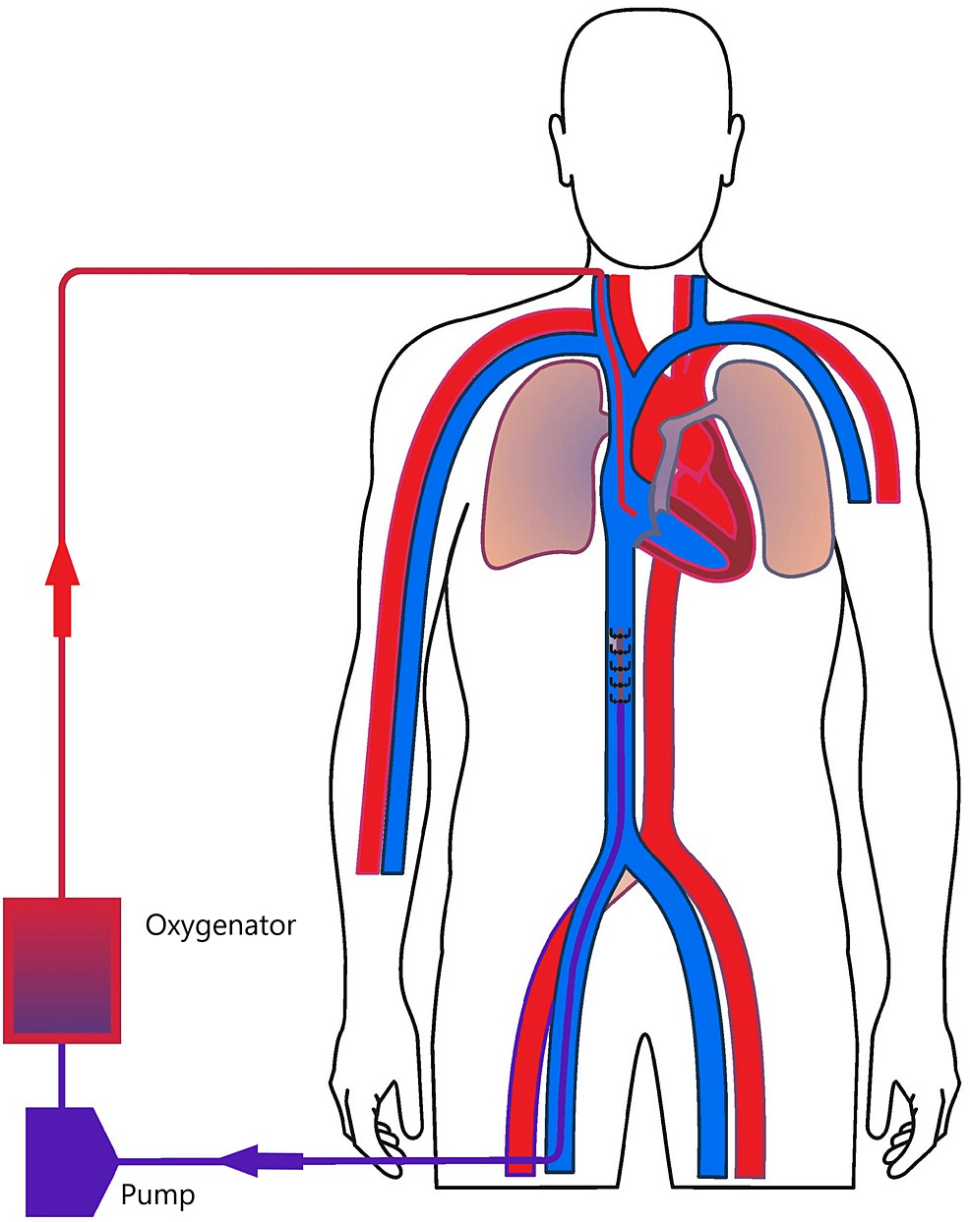
Veno-venous ECMO Diagram depicting veno-venous ECMO with right femoral vein and right internal jugular vein configuration.

**Figure 4:**
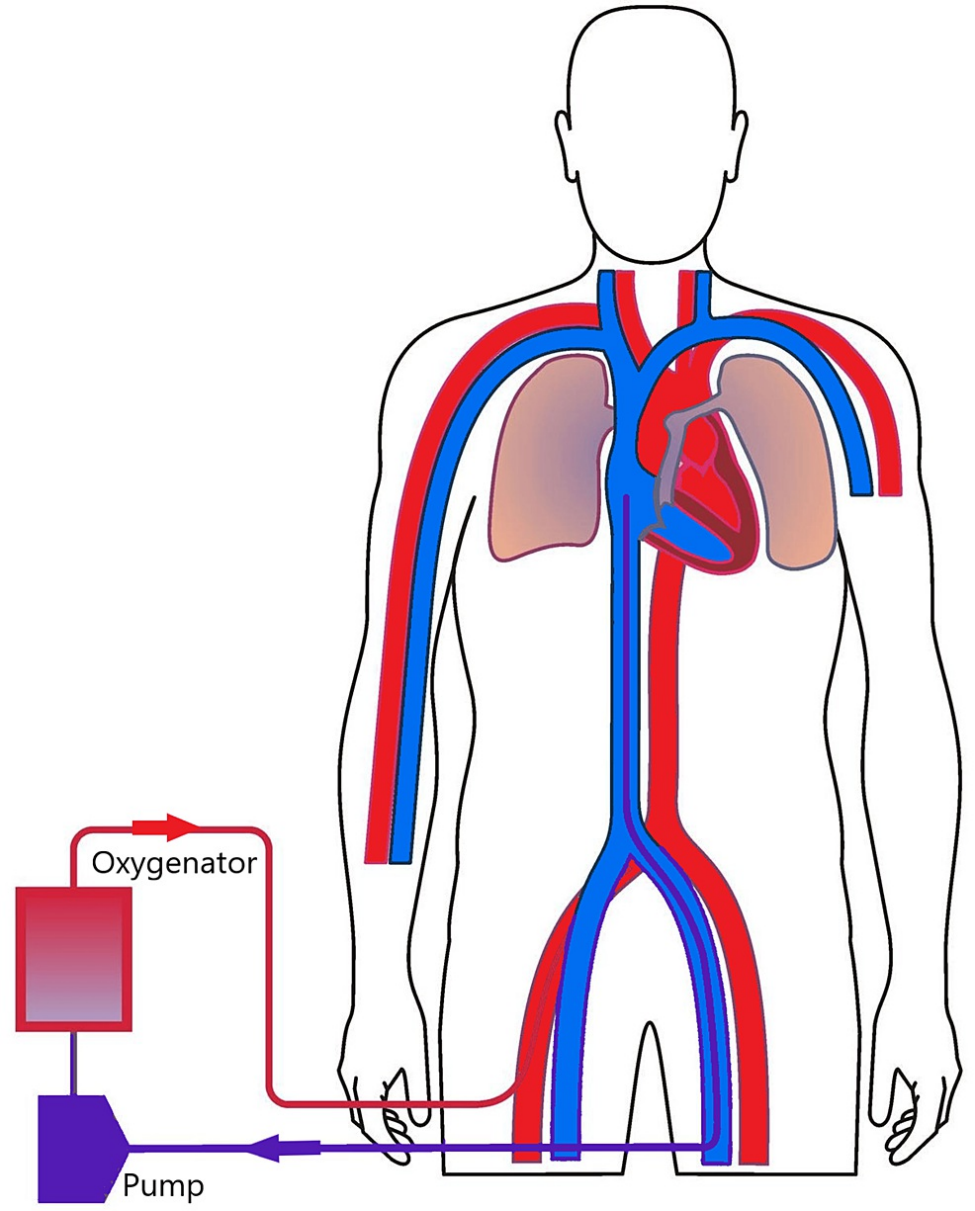
Veno-arterial ECMO Diagram depicting peripheral veno-arterial ECMO with femoral vein and femoral artery configuration.

To maximize circuit flow during VV ECMO and minimize the need for reconfiguration, larger cannulas are used after measuring the target vessel under ultrasound to ensure the cannula does not completely occlude the underlying vessel. Determining if the vessel would become completely occluded is based on the conversion of one millimeter in vessel diameter equal to three French in cannula diameter. The bedside VV ECMO cannulation strategy most commonly involves a 29-French right femoral multi-stage drainage cannula and a 23-French right internal jugular return cannula (Figure 3). If the target vessels cannot accommodate the larger cannulas, a 25-French drainage and 21-French return cannulas are typically utilized and inserted without difficulty. For VA ECMO, we opt for a smaller arterial cannula with a diameter of 15 or 17-French and a 24-French venous cannula (Figure 4). The strategy to place smaller arterial cannulas along with the placement of a 6-French distal reperfusion cannula is intended to minimize the risk of limb ischemia.

**Figure 5:**
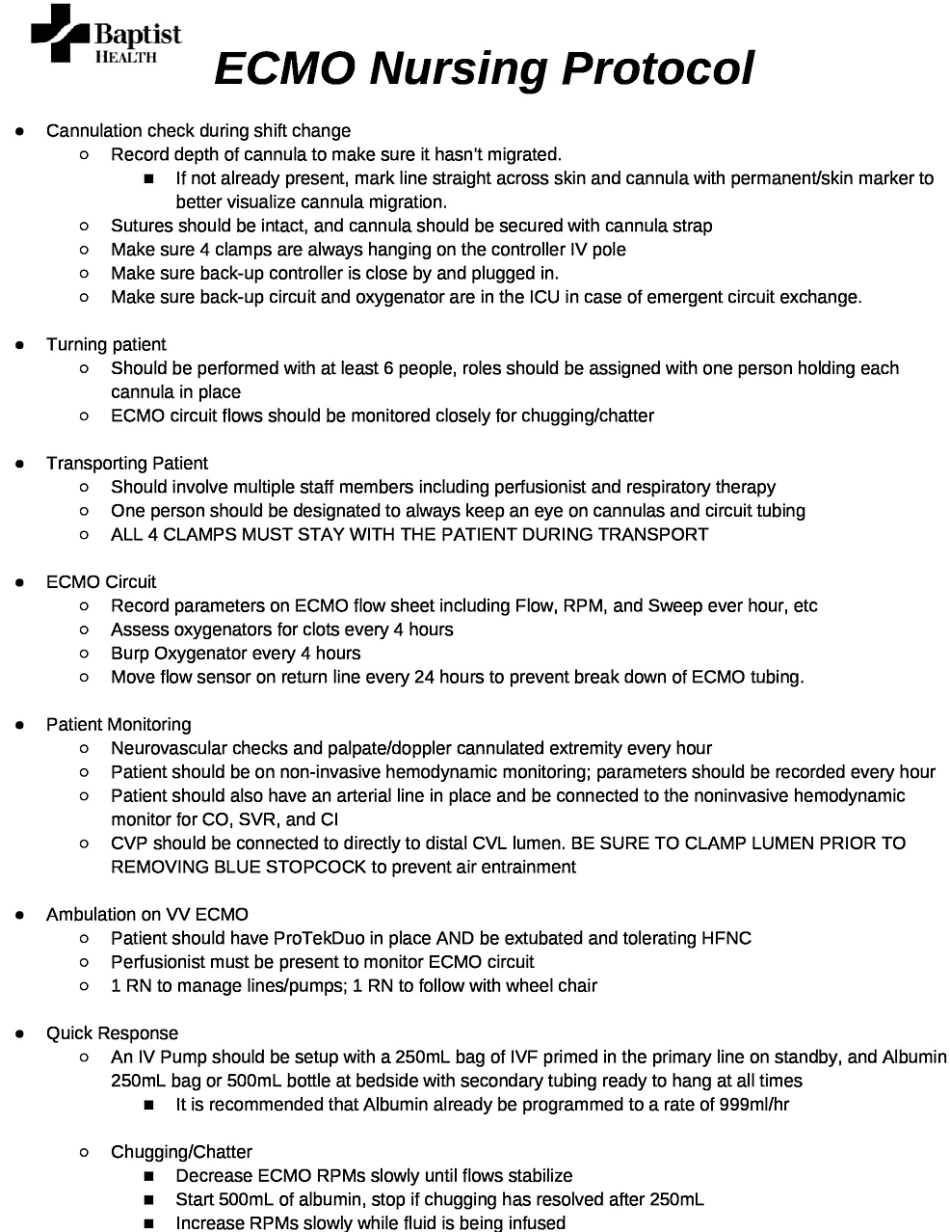
ECMO Nursing Protocol ICU: intensive care unit, CO: cardiac output, SVR: systemic vascular resistance, CI: cardiac index, CVL: central venous line, HFNC: high flow nasal cannula, RN: registered nurse, IVF: intravenous fluids, RPM: revolutions per minute

**Figure 6:**
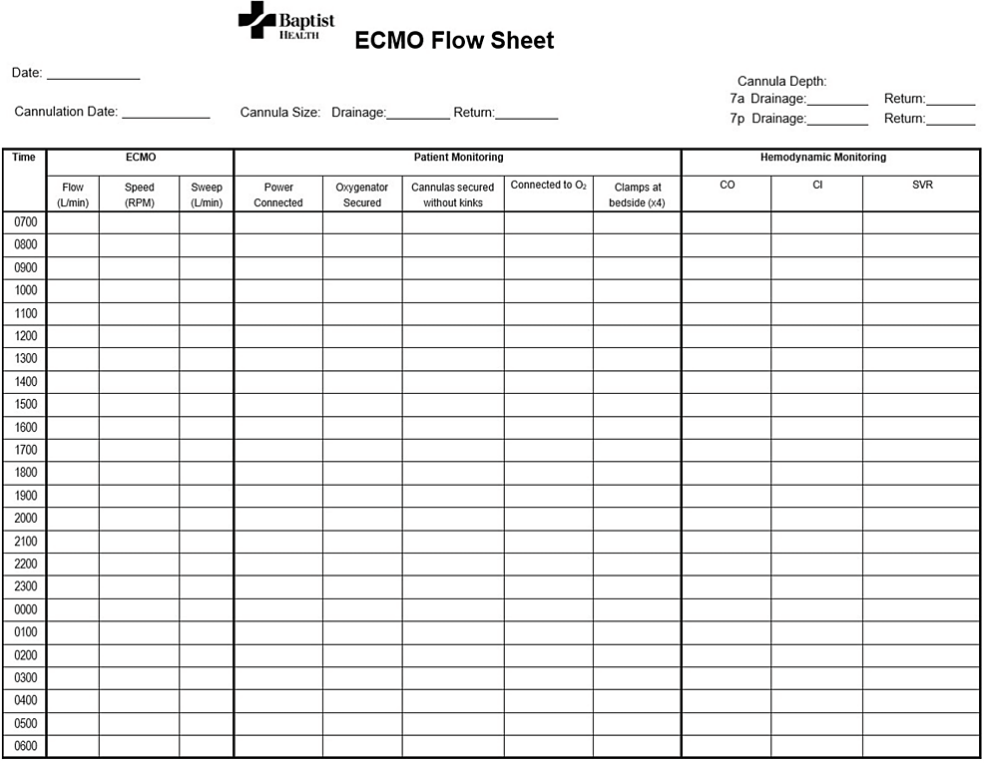
ECMO Flow Sheet RPM: revolution per minute, CO: cardiac output, CI: cardiac index, SVR: systemic vascular resistance

To implement a nurse-driven model without prior ECMO experience, it was necessary to establish a protocol that covers critical aspects of nursing care, including patient ambulation, transport, turning, circuit parameter documentation, and chatter management, among others (Figures 5-6). The protocol was then reviewed by ECMO nurses, who provided input for clarification and modification before it was approved by the ECMO program director.

A multi-disciplinary task force was formed to incorporate protocols and order sets in the electronic medical record. The task force consists of the ECMO program director, ECMO co-ordinator, perfusionist, director of clinical pharmacy, director of laboratory services, ICU nurse managers, and a clinical informatics specialist. During bi-monthly meetings, protocols and order sets were reviewed prior to incorporation into the electronic medical record. Order sets were developed in accordance with ELSO recommendations [17].

In addition to formal protocols, rapid stabilization steps were written on the door inside patient rooms immediately after ECMO initiation. Rapid stabilization steps were individualized based on the patient's underlying clinical condition. These steps are to be followed in cases of critical circuit malfunction when a physician is not immediately available at the bedside. For example, for patients on VV ECMO, the critical hypoxemia protocol involves activating emergency ventilator settings after assessing the ECMO circuit for sweep disconnection. Rapid stabilization steps are reviewed with the bedside nurse and ECMO nurse to ensure prompt and effective implementation.

Initial training and education

Staff underwent a comprehensive 12-hour training program consisting of modules, lectures, and wet labs before managing the first ECMO patient. The ELSO ECMO 101 online module was utilized as a primer before formal lectures were introduced. To allow for greater flexibility, lectures shifted from in-person to a virtual format.

A vital component of initial training and education occurred at the bedside during the management of the first three patients. A designated LivaNova representative provided additional in-person bedside support and guidance to ECMO nurses as they gained experience. During daily rounds, the ECMO program director presented the ECMO nurses with hypothetical clinical scenarios to assess and correct gaps in knowledge. The ECMO program director also observed and provided feedback on aspects of nursing care that were considered high risk for circuit disruption, such as patient turning and transport.

Continuing training and education

To complement bedside teaching, a website was developed aimed at increasing the efficiency and flexibility of ECMO education. The content is regularly updated to include recorded lectures, ELSO guidelines, the ELSO Red Book, the ECMO Specialist Training Manual, and ECMO research articles. In addition to the website, quarterly simulation sessions were added to expose learners to rapidly deteriorating ECMO patients. Simulations are conducted over a two-day period divided into two-hour sessions. With the expansion of the ECMO nursing team, simulations enabled the ECMO program director to observe the stabilization capabilities of the nurses and determine their readiness to begin managing ECMO patients.

Patient management

Personnel involved in the management of ECMO patients include an intensivist, cardiothoracic surgeon, ECMO nurse, bedside nurse, perfusionist, and clinical pharmacist. One intensivist takes responsibility for 24-hour coverage, manages all aspects of patient care, develops a daily care plan, and reviews rapid stabilization protocols during rounds. Cardiothoracic surgery provides additional recommendations on patient care in collaboration with the managing intensivist. Nurses who are assigned to ECMO patients have been vetted by the ECMO medical director and are skilled at stabilizing critically ill patients and executing prescribed management plans. The perfusionist monitors the ECMO circuit daily and obtains circuit blood gases to assess oxygenator function when necessary. In situations where a surgical procedure is needed, the perfusionist is also responsible for managing the circuit and communicating any issues with the operating room team. Anticoagulation and potential drug-circuit interactions are closely monitored by a critical care pharmacist during the day and an emergency medicine pharmacist overnight.

Specific management strategies were implemented for both VV and VA ECMO cases in attempts to mitigate adverse events. For VV ECMO patients, an airway pressure release ventilation (APRV) strategy was implemented based on the approach used in the Extracorporeal Membrane Oxygenation for Severe Acute Respiratory Distress trial [16]. The rationale for this strategy was to maintain enough lung recruitment to implement the emergency ventilator setting as a temporizing measure in the event of unexpected circuit failure. For VA ECMO patients with left ventricular failure, an “ECPELLA” strategy with the insertion of an Impella CP left ventricular assist device to allow for pre-emptive left ventricular venting.

## Results

Between December 2021 and March 2023, 11 patients were cannulated for ECMO. Nine patients were cannulated for VV ECMO and two for VA ECMO (Table 1). Of the two VA ECMO patients, one required central cannulation after failure to come off of cardiopulmonary bypass. Three patients required transfer to a large academic medical center for treatment that was beyond the capabilities of our facility and were followed for inclusion in the study. The mean age was 38 years old, with a standard deviation of 15.9. Notably, the age range of the patients who received ECMO varied significantly with the youngest being 25 years old and the oldest being 69 years old. Hypertension and morbid obesity were the two most common pre-existing medical conditions observed in 64% and 55% of patients, respectively. Although acute respiratory distress syndrome was the most frequently observed ECMO indication, we encountered a unique case of a patient with pulmonary edema secondary to acute aortic insufficiency requiring emergent VV ECMO cannulation to facilitate stabilization and transport to the operating room.

**Table 1:** Patient Demographics SD: standard deviation, ECMO: extracorporeal membrane oxygenation, ARDS: acute respiratory distress syndrome

Characteristic	Patient (N=11)
Age, mean (SD)	38 (15.9)
Race-no (%)	
Black	5 (45.5)
White	6 (54.5)
Gender-no (%)	
Male	7 (63.6)
Female	4 (36.4)
Past Medical-no (%)	
Hypertension	7 (63.6)
Diabetes	2 (18.2)
Morbid Obesity	6 (54.5)
Coronary Artery Disease	2 (18.2)
ECMO Modality- no (%)	
Veno-venous	9 (81.8)
Veno-arterial	2 (18.2)
ECMO Indication- no (%)	
Bacterial Pneumonia, ARDS	4 (45.5)
COVID-19 Pneumonia, ARDS	3 (27.3)
Air Embolism, ARDS	1 (9.1)
Aortic Valve Endocarditis, Pulmonary Edema	1 (9.1)
Pulmonary Embolism, Cardiogenic shock	1 (9.1)
Post cardiotomy, Cardiogenic shock	1 (9.1)

One transferred and seven non-transferred patients survived hospital discharge. Overall, survival was 73% (n=8), with seven patients being discharged to home and one to an inpatient rehabilitation facility (Table 2). The median duration of ECMO support was 12 days (IQR=4-16). Among the three patients who did not survive, two were cannulated for VV ECMO and one for VA ECMO. The only major adverse event was limb ischemia in a VA ECMO patient.

**Table 2:** Patient Outcomes IQR: interquartile range, ECMO: extracorporeal membrane oxygenation

Outcomes	Patient (N=11)
Major Adverse Events-no (%)	
Limb Ischemia	1 (9.1)
Days on ECMO, median (IQR)	12 (4-16)
Transfer-no (%)	3 (27.2)
Survival to hospital discharge- no (%)	8 (72.7)

## Discussion

Over the past 14 months, the program was able to demonstrate the safe and effective implementation of a non-tertiary ECMO program. Despite the inexperience and limited resources, survival to hospital discharge was 73%, and only one patient had a major adverse event. These findings are comparable to, and even surpass, similar studies at larger referral centers with new ECMO programs [9,14,18].

Adherence to established guidelines, as well as a tailored approach to patient care and staff training, may provide the foundation for a successful community hospital-based ECMO program. It is also important to simplify ECMO management and provide extra support to bedside staff. These principles should be taken into consideration in the planning and early developmental phases of the program.

A model focused on incremental patient complexity, strategies that optimize patient safety, and high-yield training can lead to outcomes similar to those at larger ECMO programs. A stepwise approach that began with VV ECMO cases allowed ECMO nurses to develop confidence, experience, and knowledge prior to moving forward to the more challenging VA ECMO phase of the program. Proactive management strategies, such as the insertion of an Impella left ventricular assist device, the use of smaller arterial cannulas, checklists, and emergency protocols, have been shown to improve patient safety [19-22]. These strategies were utilized effectively and could prove useful for other community hospital ECMO programs with limited resources.

As the program evolved, ECMO education was optimized by launching a website and conducting high-stress simulations to complement bedside teaching. Similar to other programs, the direct observation of patient stabilization and application of ECMO knowledge was useful in determining readiness for patient care [23]. For low-volume medical centers, maximizing education can help staff remain prepared for future ECMO cases.

The COVID-19 pandemic and other respiratory viruses will continue to pose challenges to healthcare systems worldwide. In times of high patient volume and system stress, larger medical centers may not be able to provide ECMO support to smaller regional hospitals. Without a framework for developing an ECMO program, patients at smaller resource-limited hospitals will not have access to a potentially life-saving therapy.

Although this small patient cohort may limit the reproducibility of these results, it adds to the current literature and provides guidance specific to non-tertiary medical centers. Further studies are needed to solidify the best approach and model for the development of community hospital ECMO programs.

## Conclusions

The safe implementation of an ECMO program at a non-tertiary hospital with inexperienced staff and limited resources is challenging but feasible. Survival rates that exceed those of larger tertiary hospitals are achievable with a proactive approach that emphasizes adherence to established guidelines, effective training techniques, patient management protocols, and strategies to mitigate adverse events and limit resource utilization.
